# Unmet social needs and diverticulitis: a phenotyping algorithm and cross-sectional analysis

**DOI:** 10.1093/jamia/ocae238

**Published:** 2025-03-14

**Authors:** Thomas E Ueland, Samuel A Younan, Parker T Evans, Jessica Sims, Megan M Shroder, Alexander T Hawkins, Richard Peek, Xinnan Niu, Lisa Bastarache, Jamie R Robinson

**Affiliations:** Vanderbilt University School of Medicine, Nashville, TN 37232, United States; Division of General Surgery, Vanderbilt University Medical Center, Nashville, TN 37232, United States; Department of Biomedical Informatics, Vanderbilt University Medical Center, Nashville, TN 37232, United States; Department of Pediatric Surgery, Vanderbilt University Medical Center, Nashville, TN 37232, United States; Vanderbilt University School of Medicine, Nashville, TN 37232, United States; Division of General Surgery, Vanderbilt University Medical Center, Nashville, TN 37232, United States; Division of General Surgery, Vanderbilt University Medical Center, Nashville, TN 37232, United States; Division of Gastroenterology, Hepatology, and Nutrition, Vanderbilt University Medical Center, Nashville, TN 37232, United States; Department of Biomedical Informatics, Vanderbilt University Medical Center, Nashville, TN 37232, United States; Department of Biomedical Informatics, Vanderbilt University Medical Center, Nashville, TN 37232, United States; Department of Biomedical Informatics, Vanderbilt University Medical Center, Nashville, TN 37232, United States; Department of Pediatric Surgery, Vanderbilt University Medical Center, Nashville, TN 37232, United States

**Keywords:** diverticular disease, social determinants of health, phenotyping algorithm

## Abstract

**Objective:**

To validate a phenotyping algorithm for gradations of diverticular disease severity and investigate relationships between unmet social needs and disease severity.

**Materials and Methods:**

An algorithm was designed in the *All of Us* Research Program to identify diverticulosis, mild diverticulitis, and operative or recurrent diverticulitis requiring multiple inpatient admissions. This was validated in an independent institution and applied to a cohort in the *All of Us* Research Program. Distributions of individual-level social barriers were compared across quintiles of an area-level index through fold enrichment of the barrier in the fifth (most deprived) quintile relative to the first (least deprived) quintile. Social needs of food insecurity, housing instability, and care access were included in logistic regression to assess association with disease severity.

**Results:**

Across disease severity groups, the phenotyping algorithm had positive predictive values ranging from 0.87 to 0.97 and negative predictive values ranging from 0.97 to 0.99. Unmet social needs were variably distributed when comparing the most to the least deprived quintile of the area-level deprivation index (fold enrichment ranging from 0.53 to 15). Relative to a reference of diverticulosis, an unmet social need was associated with greater odds of operative or recurrent inpatient diverticulitis (OR [95% CI] 1.61 [1.19-2.17]).

**Discussion:**

Understanding the landscape of social barriers in disease-specific cohorts may facilitate a targeted approach when addressing these needs in clinical settings.

**Conclusion:**

Using a validated phenotyping algorithm for diverticular disease severity, unmet social needs were found to be associated with greater severity of diverticulitis presentation.

## Introduction

Social determinants of health (SDOH) are the “conditions and environments in which people are born, live, learn, work, play, worship, and age.”^1^ The World Health Organization (WHO) distinguishes between structural determinants (ie, socioeconomic status) and intermediary determinants (ie, food insecurity), and highlights intermediary determinants as the direct agents impacting vulnerability to poor health states.^2^ Despite known implications for medical outcomes, intermediary determinants and individual-level information are sparse among clinical databases compared to area-level social deprivation metrics.^3^ Through collection of area-level social indices, structural determinants, intermediary determinants, and clinical records, the *All of Us* Research Program provides unique opportunities to probe underlying drivers of SDOH.^4^

Diverticular disease refers to outpouchings in a weakened intestinal wall.^5^ There is tremendous variation in the clinical disease across the severity spectrum from asymptomatic diverticulosis to emergent diverticulitis with sequalae such as abscess, macroperforation, obstruction, stricture, or fistula.^6^ Prior research has described a phenotyping algorithm to separate cases of any diverticular disease from controls with no diverticular disease, but did not capture distinctions of disease severity.^7^ Thus, there is no validated approach approximating disease severity from the electronic medical record. The International Classification of Diseases, Tenth Revision, Clinical Modification (ICD-10-CM) coding system includes specifications for uncomplicated and complicated disease, but positive predictive values (PPV) have been variable and as low as 0.67.^8^

This study aimed to validate a phenotyping algorithm that captures gradations of diverticular disease severity and to describe social barriers in this cohort. We examined the distribution of individual-level needs across an area-level deprivation index, and performed adjusted analysis with selected barriers: food insecurity, housing instability, and delaying or inability to afford care.^9–12^ We hypothesized that patients with unmet social needs would be more likely to present with more severe diverticular disease.

## Methods

### Sub-phenotyping algorithm

A rule-based algorithm was designed in the *All of Us* Research Program through the Cohort Builder tool combining diagnostic codes, procedural codes, imaging codes, settings of care, and temporal relationships (Figure S1; Supplementary Material).^13^ The first portion of the algorithm adapted the previously validated approach for separating cases of diverticular disease from controls of no diverticular disease.^7^ Cases of any diverticular disease were identified through an ICD-9 or ICD-10-CM code for colonic diverticular disease within 7 days after an abdominal computed tomography scan or lower gastrointestinal endoscopic procedure. The second portion of the algorithm aimed to differentiate limited from more severe forms of the disease, categorizing diverticular disease into 3 distinct groups. The Diverticulosis group comprised cases who were only assigned diverticulosis codes. The Mild Diverticulitis group included patients with only outpatient encounters or no greater than 1 inpatient encounter for diverticulitis. The Operative or Recurrent Inpatient Diverticulitis group identified patients with more than 1 inpatient admission for diverticulitis or a procedure (colectomy, percutaneous drain, and intestinal fistula repair) performed for diverticulitis.

Validation of the algorithm was performed in an independent deidentified clinical records repository with over 3.6 million distinct participants, the Vanderbilt Synthetic Derivative.^14^ For controls and each sub-phenotype, 200 charts were sampled and reviewed by a content expert who was blinded to the algorithm assignment status. From the total 800 patients included in the validation, congruence between the algorithm and manual review was assessed through PPV and Negative Predictive Value (NPV) scores. Inter-rater reliability of the assignments by manual review was assessed using a sample of 400 patients reviewed by 2 additional content experts through Cohen’s kappa coefficient and percentage of differential classification.^15^ Discrepancies between reviewers were resolved through iterative review and discussion to provide a consensus. In addition to examining the distribution of complicated versus uncomplicated disease across our severity groups, we assessed the reliability of a code-only approach by manually reviewing 150 patients who were assigned codes specific for complicated diverticulitis and quantifying the frequency of true “complicated diverticulitis” in this group.

### Social determinants of health

We performed a cross-sectional analysis of adult participants in the *All of Us* Research Program Controlled Tier Version 7 dataset.^4^ Using the phenotyping algorithm, we identified a cohort of diverticular disease cases occurring within 5 years of initial survey completion. Availability of data for social needs (food insecurity, housing instability, delayed or can’t afford care) was compared across diverticular disease severity groups. Then, participants with unavailable data for social needs were excluded for the remainder of the analysis. Sociodemographic features were extracted from survey responses, clinical records, and the pre-computed Brokamp Deprivation Index.^16^

Social variables were grouped according to the United States Department of Health and Human Services’ Healthy People 2030 framework (Supplementary Material).^1^ The “Economic Stability” domain was represented with Hunger Vital Sign scores, annual income, and housing instability.^17^ “Education Access and Quality” included the highest level of education. “Healthcare Access and Quality” consisted of “Delayed” or “Can’t Afford Care” survey items, health insurance, and the Brief Health Literacy Screen.^18^ “Neighborhood and Built Environment” was represented with the Ross-Mirowsky Perceived Neighborhood Physical Disorder Subscale and the Housing Quality item.^19^ “Social and Community Context” included the Modified Medical Outcomes Study Social Support Survey and English proficiency.^20^

### Statistical analysis

We performed descriptive statistics using median and interquartile range (IQR) for continuous variables and frequency for categorical variables. Baseline characteristics across diverticular disease severities were compared with Kruskal-Wallis rank sum test for continuous variables and Pearson’s chi-squared test for categorical variables. When comparing individual-level social barriers with area-level quintiles, we transformed scored surveys to binary variables through a top box conversion. A top box score is achieved only with the most favorable responses to each survey question, while all other combinations of responses are assigned a non-top box score (Supplementary Material). For each area-level index quintile, we divided counts of an individual-level social barrier in that quintile by the total counts of the social variable across all quintiles. A fold enrichment was obtained by dividing the proportion in the fifth (most deprived) quintile by the first (least deprived) quintile.

For adjusted modeling, the most severe group (Operative and Recurrent Diverticulitis) was compared to the least severe group (Diverticulosis) with respect to unmet social needs: food insecurity, housing instability, and delaying or inability to afford care. This was informed by prior evaluations of impact with SDOH interventions^9–11^^,^^21–23^ as well as qualitative analyses of diverticulitis patients with severe presentations.^24^^,^^25^ These factors are also intermediary determinants that represent needs for which local resources are often available and could be plausibly addressed in a clinical setting. A binary indicator of any unmet social need was included as a covariate with age, sex at birth, Charlson Comorbidity Index (CCI),^26^^,^^27^ body mass index (BMI), annual income, and highest education level in multivariable logistic regression with diverticulitis severity as the outcome. Interaction terms were included for the unmet social need term with annual income and with highest level of education. Multicollinearity was assessed through variance inflation factors, and covariates with less than 20% missingness were imputed through bagged trees models. Filters for variable levels with near-zero variance or absolute correlations >0.90 were applied during model development. Sensitivity analyses were performed for adjusted models that reduced the inclusion time window between survey completion and date of the procedure. Statistical analysis was performed using R version 4.2.3 in an approved Researcher Workbench workspace. To synthesize findings and facilitate public engagement with results, a Public Summary Guideline was created and linked in the study’s description on the *All of Us* Research Program website.

## Results

### Phenotyping algorithm

**Figure 1.**
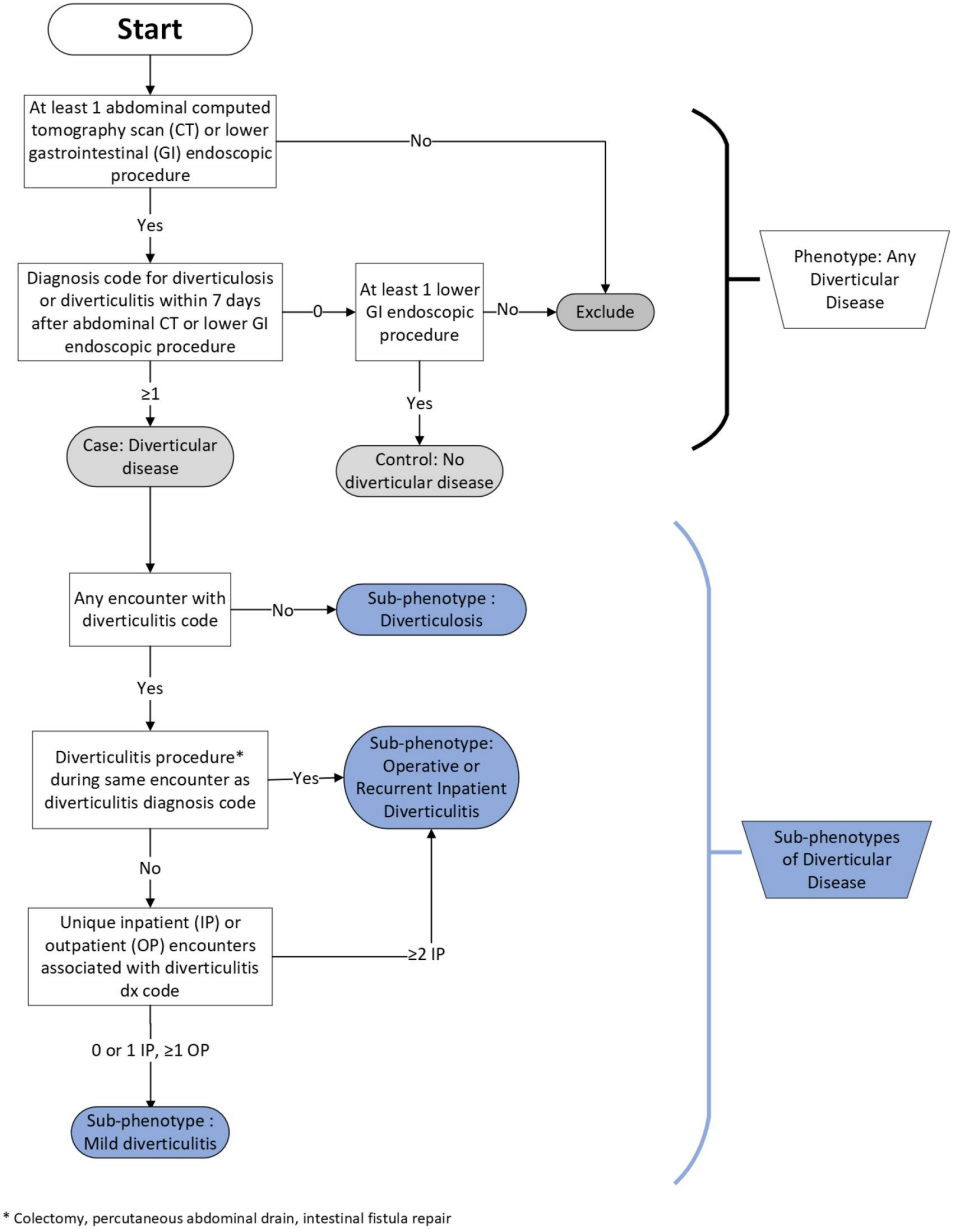
Sub-phenotyping algorithm for diverticular disease severity.

The phenotyping algorithm developed to classify severity of diverticular disease performed well in the validation cohort of the Vanderbilt Synthetic Derivative, as the algorithm’s per-class PPV ranged from 0.87 (Mild Diverticulitis) to 0.97 (Control) and NPV ranged from 0.97 (Operative or Recurrent Inpatient Diverticulitis) to 0.99 (Control) (Figure 1; Table 1). Inter-rater reliability was in the “almost perfect” range (Cohen’s kappa 0.95), and the percentage of differential classification by class was 2.4% for Control, 1.0% for Diverticulosis, 2.2% for Mild Diverticulitis, and 9.6% for Operative or Recurrent Inpatient Diverticulitis.^15^ The most common reason for misassignment was an inability to account for outside hospital records mentioned in clinical notes (Supplementary Material). When assessing efficacy of codes alone for phenotyping accuracy in our cohort, we found that among patients who were assigned codes for complicated diverticulitis, only 70% met criteria for complicated diverticulitis on manual review (Supplementary Material). We evaluated the Operative or Recurrent Inpatient Diverticulitis group to compare our algorithm to the clinical standard of classification through uncomplicated and complicated codes. In this group, 89% of the operative cases had confirmation of complicated disease as opposed to 37% of the recurrent inpatient cases.

**Table 1.** Algorithm validation.

Sub-phenotype^a^	PPV	NPV
Control	0.97	0.99
Diverticulosis	0.96	0.98
Mild diverticulitis	0.87	0.97
Operative or recurrent inpatient diverticulitis	0.91	0.97

a Algorithm performance evaluated relative to blinded manual chart review of 200 patients from each sub-phenotype group.

Abbreviations: NPV = negative predictive value; PPV = positive predictive value.

### Distribution of SDOH

Individuals with missing information about unmet social needs had greater rates of Operative or Recurrent Inpatient Diverticulitis (5.7% versus 4.1%; *P* < .01), annual income less than 50k (55% versus 39%; *P* < .01), and education level less than high school (13% versus 5.7%; *P* < .01) (Table 2). Among participants with available data on social needs, there were 18 972 controls without diverticular disease, 6556 cases of Diverticulosis, 1337 cases of Mild Diverticulitis, and 335 cases of Operative or Recurrent Inpatient Diverticulitis (Table 3). In order of increasing severity from controls without diverticular disease to Operative or Recurrent Inpatient Diverticulitis, there were differences in sex at birth (63% versus 55% versus 63% versus 64%; *P* < .01), PROMIS-10 Global Physical T-scores (median [IQR] 48 [42, 54] versus 48 [42, 54] versus 45 [40, 54] versus 45 [37, 51]; *P* < .01) and PROMIS-10 Mental T-scores (median [IQR] 51 (44, 56) versus 51 [44, 56] versus 51 [44, 56] versus 48 [41, 53]; *P* < .01). The highest proportion of unmet social needs was in the Operative or Recurrent Inpatient Diverticulitis group (50%) relative to the Mild Diverticulitis group (45%) or the Diverticulosis group (38%).

**Table 2.** Missingness of unmet social needs.

Characteristic	Available Information (*N* = 8228)	Missing information (*N* = 6892)	*P*-value
Sex at birth			.50
Female	4576 (57%)	3877 (57%)	
Male	<3510	<2870	
Other	<20	<20	
Age at CDR cutoff (years)	68 (60, 74)	69 (61, 76)	<.001
BMI (kg/m^2^)	30 (26, 35)	30 (26, 35)	.02
Charlson comorbidity index	3 (2, 5)	4 (2, 6)	<.001
Diverticulitis phenotype			<.001
Diverticulosis	6556 (80%)	5328 (77%)	
Mild diverticulitis	1337 (16%)	1170 (17%)	
Operative or recurrent inpatient diverticulitis	335 (4.1%)	394 (5.7%)	
Annual income			<.001
Less than 50k	2749 (39%)	2679 (55%)	
50-100k	1892 (27%)	1110 (23%)	
More than 100k	2351 (34%)	1077 (22%)	
Highest education level			<.001
Less than high school degree or equivalent	460 (5.7%)	836 (13%)	
High school degree or equivalent	1058 (13%)	1535 (23%)	
Some college, college degree, or advanced degree	6537 (81%)	4259 (64%)	

Values represent *n* (%) or Median (IQR). Missingness counts omitted and values less than or equal to 20 are not shown to maintain compliance with *All of Us* Data and Statistics Dissemination Policy. *P* values represent Wilcoxon rank sum test; Pearson’s Chi-squared test.

Abbreviations: BMI = body mass index; CDR = curated data repository.

**Table 3.** Characteristics across diverticular disease sub-phenotype groups.

Characteristic	Control: no diverticular disease (*N* = 18 972)	Diverticulosis (*N* = 6556)	Mild diverticulitis (*N* = 1337)	Operative or recurrent inpatient diverticulitis (*N* = 335)	*P*-value
Sex at birth					<.001
Female	11 805 (63%)	3542 (55%)	823 (63%)	211 (64%)	
Male	6786 (36%)	<2910	<490	<420	
Other	<20	<20	<20	<20	
Age at CDR cutoff (years)	62 (53, 70)	68 (60, 75)	67 (57, 74)	66 (58, 73)	<.001
BMI (kg/m^2^)	28 (25, 33)	30 (26, 35)	31 (27, 36)	30 (27, 35)	<.001
Charlson comorbidity index	2 (1, 4)	3 (2, 5)	3 (2, 5)	4 (2, 6)	<.001
Annual income					<.001
Less than 50k	6302 (39%)	2099 (38%)	528 (46%)	122 (45%)	
50-100k	4212 (26%)	1523 (27%)	299 (26%)	70 (26%)	
More than 100K	5834 (36%)	1959 (35%)	310 (27%)	82 (30%)	
Employment					<.001
Out of work	1127 (6.1%)	302 (4.7%)	79 (6.1%)	<20	
Employed	8875 (48%)	2368 (37%)	485 (37%)	133 (40%)	
Other	8448 (46%)	3749 (58%)	739 (57%)	179 (54%)	
Hunger vital sign positive screen	1335 (13%)	417 (11%)	99 (14%)	32 (20%)	.001
Highest education level					<.001
Less than high school degree or equivalent	853 (4.6%)	357 (5.6%)	80 (6.1%)	23 (7.0%)	
High school degree or equivalent	2365 (13%)	783 (12%)	229 (18%)	46 (14%)	
Some college, college degree, or advanced degree	15 266 (83%)	5284 (82%)	995 (76%)	258 (79%)	
Insurance: none or not accepted	1805 (9.5%)	509 (7.8%)	125 (9.4%)	25 (7.5%)	<.001
Brief health literacy screen	15 (13, 15)	15 (13, 15)	14 (13, 15)	15 (13, 15)	.01
Delayed or can’t afford care	4281 (26%)	1334 (24%)	336 (30%)	79 (29%)	<.001
Housing quality concern	1875 (19%)	603 (17%)	113 (17%)	28 (19%)	.10
Housing instability	3953 (21%)	1207 (19%)	304 (23%)	89 (27%)	<.001
Perceived physical neighborhood disorder scale	1.50 (1.17, 2.00)	1.50 (1.17, 2.00)	1.50 (1.17, 2.00)	1.50 (1.00, 1.83)	.40
Modified medical outcomes study social support survey	4.13 (3.25, 4.75)	4.13 (3.13, 4.75)	4.00 (3.00, 4.75)	4.00 (3.00, 4.88)	.11
English proficiency barrier	112 (1.1%)	48 (1.3%)	<20	<20	.09
PROMIS-10 Global Mental score (T-score)	51 (44, 56)	51 (44, 56)	51 (44, 56)	48 (41, 53)	<.001
PROMIS-10 Global Physical score (T-score)	48 (42, 54)	48 (42, 54)	45 (40, 54)	45 (37, 51)	<.001
Unmet social need^a^	7963 (42%)	2480 (38%)	606 (45%)	168 (50%)	<.001

Values represent *n* (%) or Median (IQR). Missingness counts omitted and values less than or equal to 20 are not shown to maintain compliance with *All of Us* Data and Statistics Dissemination Policy. *P* values represent Kruskal-Wallis rank sum test; Pearson’s Chi-squared test.

a Unmet social need is a composite variable representing food insecurity, housing instability, and delayed or can’t afford care items.

Abbreviations: BMI = body mass index; CDR = curated data repository; PROMIS = patient-reported outcomes measurement information system.

**Figure 2.**
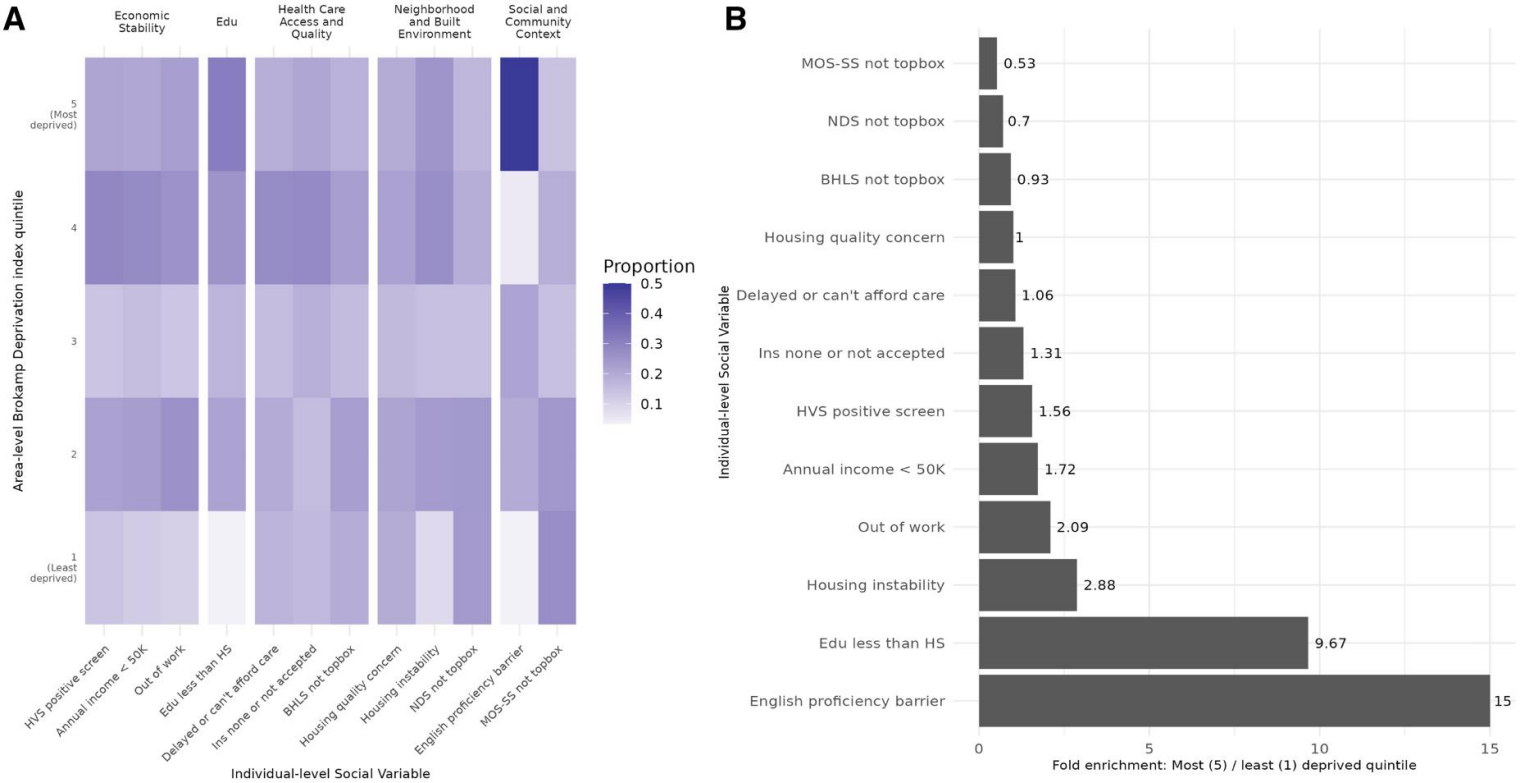
Distribution of individual-level social variables across area-level Brokamp Deprivation Index quintiles. (A) Heatmap demonstrating the proportion of social barriers within each deprivation index quintile. (B) Fold enrichment of the proportion of social barriers in the fifth (most deprived) quintile relative to first (least deprived) quintile. A top box score is achieved only with the most favorable responses to each survey question, while all other combinations of responses are assigned a non-top box score. Edu = education; VS = hunger vital sign; HS = high school; Ins = insurance; BHLS = brief health literacy screen; NDS = Ross-Mirowsky perceived neighborhood physical disorder subscale; MOS-SS = modified medical outcome study social support survey.

The area-level metric of the Brokamp Deprivation Index was not associated with severity of diverticular disease. There were 74 of 1480 (5%) cases of Operative or Recurrent Inpatient Diverticulitis in the fifth (most deprived) quintile of the Brokamp Deprivation Index compared to 73 of 1825 (4%) in the first (least deprived) quintile. However, there was substantial variability in individual-level social barriers across area-level index quintiles. The least reported social barrier was observed in the first quintile (Education Less than High School, 3.3%). The highest proportion of a self-reported social need was an English proficiency barrier (50% in the fifth quintile) (Figure 2). The proportion of individual-level social barriers was lower in the first (least deprived quintile) relative to the fifth (most deprived quintile) for the Modified Medical Outcomes Study Social Support Survey, the Ross-Mirowsky Perceived Neighborhood Physical Disorder Subscale, and the housing quality variable.

### Regression modeling

**Figure 3.**
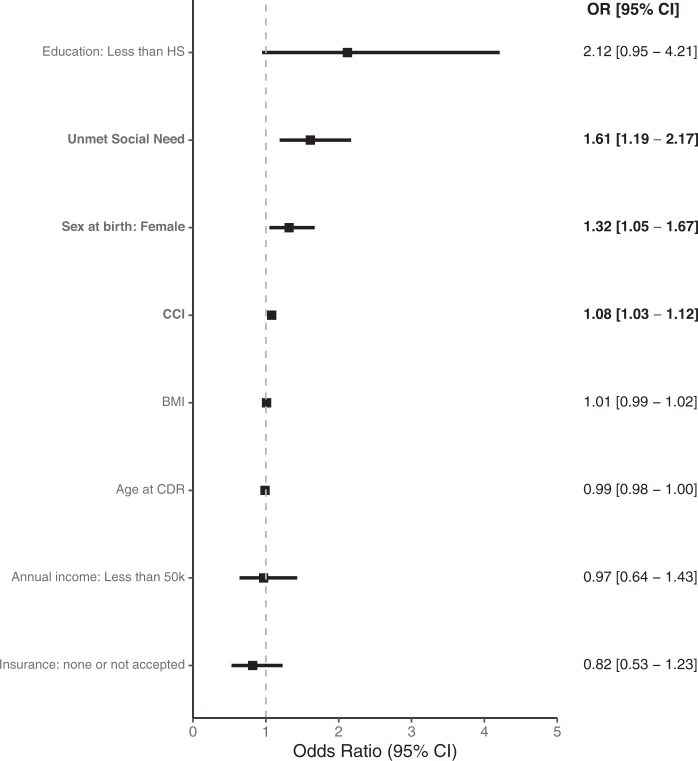
Logistic regression model for Operative or Recurrent Inpatient diverticulitis. Model covariates were age, sex at birth, BMI, Charlson Comorbidity Index, binary unmet social need variable, insurance, highest education, annual income, and interaction terms between education and unmet social need as well as income and unmet social need. OR = odds ratio; CI = confidence interval; CDR = curated data repository cutoff date; BMI = body mass index (kg/m^2^); CCI: Charlson comorbidity index; HS = high school.

**Figure 4.**
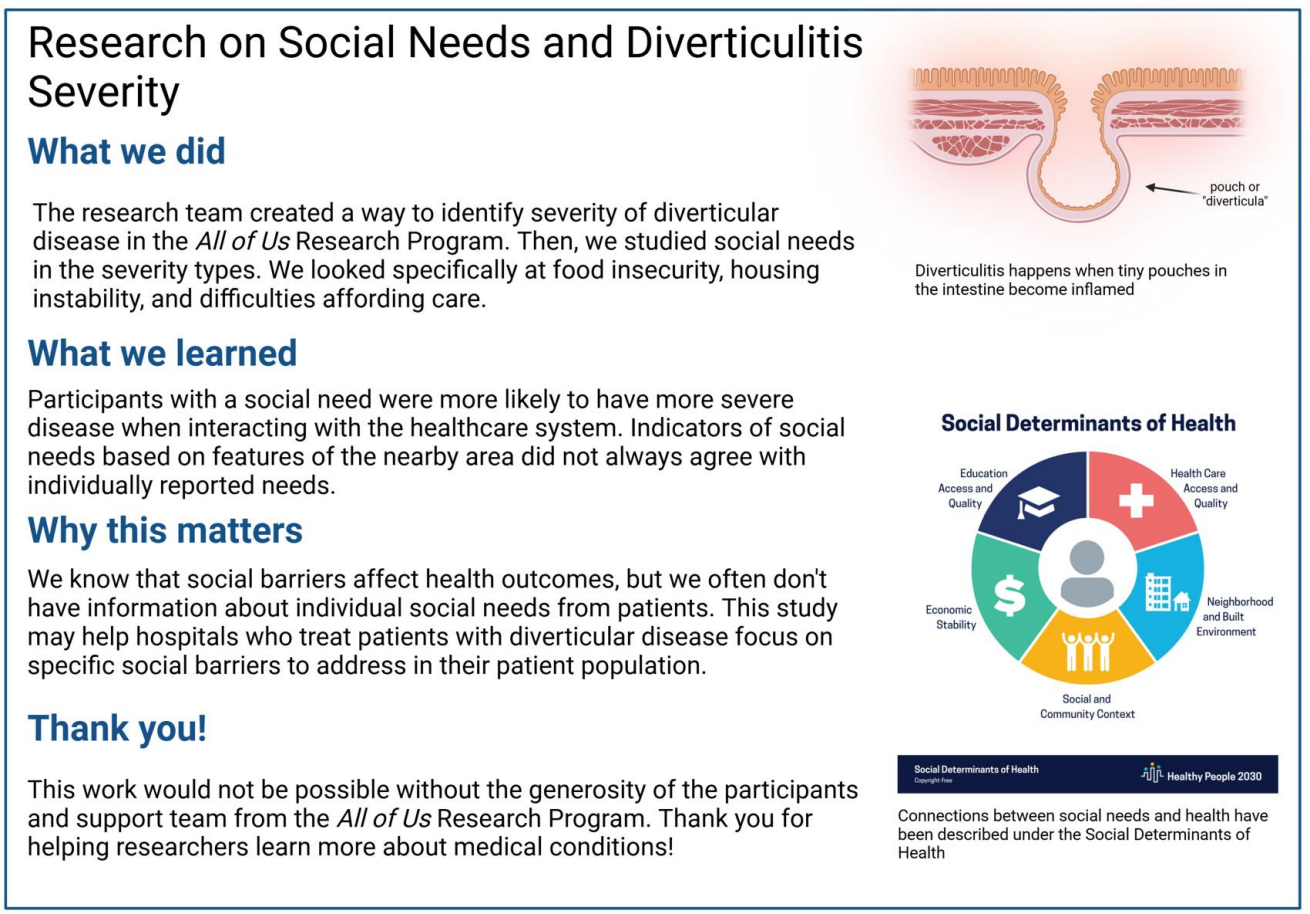
Public reporting summary.

In the adjusted model, reporting an unmet social need was associated with greater likelihood of Operative or Recurrent Inpatient Diverticulitis (reference: Diverticulosis) (OR [95% CI] 1.61 [1.19-2.17]; Figure 3). An interaction effect was detected between education less than high school and an unmet social need (*P*_interaction_ = .02), but education was not independently associated with severity of diverticulitis presentation (OR [95% CI] 2.12 [0.95-4.21]). The Public Summary Guideline to provide results to the public and participants is shown in Figure 4.

In sensitivity analyses, we reduced the inclusion window to 2.5 years between completion of the inclusion survey and date of the procedure. We also explored whether changing the inclusion survey from the earliest survey to the Healthcare Access and Utilization survey or the SDOH survey would affect results. An unmet social need remained associated with more severe presentations when narrowing the inclusion time window and when changing the inclusion survey to the Healthcare Access and Utilization questionnaire, but not when changing to SDOH as the inclusion survey (Supplementary Material).

## Discussion

This study validated a phenotyping algorithm for diverticular disease severity and found that individual-level social determinants varied across an area-level social deprivation index and across diverticular disease severities. When adjusting for indicators of socioeconomic status, an unmet social need was independently associated with greater odds of severe diverticulitis presentations.

The rule-based phenotyping algorithm reliably identified groups of Diverticulosis, Mild Diverticulitis, and Operative or Recurrent Inpatient Diverticulitis. While deviating from the clinical norm of complicated and uncomplicated diverticulitis, this framework was motivated by our hypothesis that patients with greater burden from diverticular disease would be more likely to report unmet social needs. Further, our grouping of multiple inpatient episodes with operative disease is consistent with prior work in diverticulitis.^28^ Recurrent uncomplicated diverticulitis is a burdensome disease with substantial and longitudinal impact on quality of life.^24^^,^^25^ In this group, the demands of frequent healthcare utilization may magnify the deleterious impact of social barriers, potentially beyond the degree seen in patients with a single episode of complicated diverticulitis that is treated operatively. Within our Operative or Recurrent Inpatient Diverticulitis group, the operative cases but not the recurrent inpatient cases reliably demonstrated confirmed complications on manual review (Supplementary Material). Thus, for future studies isolating complicated diverticulitis, we recommend limiting the cohort to operative cases to achieve optimal PPV. In addition, our requirement of imaging or endoscopic confirmation for cases is stricter than code-based definitions. This minimizes false positives at the expense of false negatives, as cases with imaging or procedures from other hospitals would not be identified by the algorithm. While code-based definitions are more inclusive, not all patients with diverticulitis suspected on clinical presentation have the disease. We reaffirm previous caution with sole use of diagnostic codes to identify complications.^8^

Identifying impactful social barriers tailored to a patient’s disease process may complement upstream policy efforts addressing SDOH.^9–12^^,^^21^^,^^29^ It is impractical to resolve all social needs within the context of a clinical encounter. However, an understanding of the ways in which different diseases expose underlying social stressors may illuminate a subset of needs pertinent to the disease.^30–33^ For example, Goel et al identified an association between social needs and decreased utilization of screening mammography independent from individual-level confounders or access to mammography.^33^ They hypothesized that the preventative nature of the intervention may be perceived with lower importance in the context of competing priorities related to caregiving with limited social support or flexibility to take time away from work. In diverticulitis, dietary patterns are a risk factor for severity of presentation with current recommendations favoring a balanced intake of fruits, vegetables, whole grains, and fiber.^34^^,^^35^ However, specialized high-fiber diets may not be practical for individuals with food insecurity. Similarly, barriers with accessing or affording care may raise patients’ threshold for seeking evaluation of a diverticulitis episode until bouts become more severe. Important gaps remain in the implementation of effective strategies to address social barriers in clinical settings. This task would likely benefit from greater understanding about the individual-level needs associated with severe presentations for common diseases.

Area-level deprivation indices have been crucial for establishing associations between social disadvantage and health outcomes. However, there is ongoing debate about whether to adopt area-level indices when identifying needs in clinical settings, as previous studies have reported variability between individual- and area-level measures.^36–40^ For example, Bensken et al’s national study of over 1 million community health center patients found that the best performing area-level deprivation index predicted individual social needs with an accuracy of only 0.68.^36^ In our comparison across area-level deprivation quintiles, individual social barriers were concentrated among higher quintiles. However, this pattern was not uniform, and 6 of the 12 variables had a fold enrichment of less than 1.5 in the fifth (most deprived) quintile relative to the first (least deprived). This finding that an area-level index did not fully capture the landscape of individual needs has implications for clinics or hospitals attempting to identify social needs in their patients. It also highlights the value of prospective research databases with individual-level social variables to inform the design of clinical initiatives.

In the WHO framework, the primary action of socioeconomic status is to generate inequitable distribution of downstream intermediary determinants, which in turn create vulnerabilities to poorer health states.^2^ While existing studies have linked socioeconomic indicators and diverticulitis outcomes,^41–44^ our study’s adjusted analysis focused on intermediary determinants: food insecurity, housing instability, or barriers to accessing care. The association between an unmet social need and disease severity agrees with prior examinations in other diseases.^9^^,^^11^ Further, the interaction between education and an unmet social need provides empiric support for the conceptual distinction between structural and intermediary determinants. Importantly, in our cohort, patients with missing information about social needs were more likely to have lower levels of income and education. Thus, even a data collection platform dedicated to improving representation did not eliminate the widespread pattern of nonuniform missingness across socioeconomic lines.^45–47^ As this applies not only to availability in research databases but also to capturing needs in clinical settings, additional work is needed to optimize data collection specifically for individuals with indicators of lower socioeconomic status.

The *All of Us* Research Program provides an innovative means of universal engagement through their Discover online portal, where a Research Projects Directory describes ongoing projects and a Publications section displays links to peer-reviewed articles. However, manuscripts may not be accessible to those without journal subscriptions and comprehension of contents written for an academic readership may be limited. To improve public dissemination of research findings, we created a Public Summary Report informed by available dissemination toolkits.^48^^,^^49^ In the study’s Research Projects Directory entry, we included a link to the Public Summary with our immediate goal being to facilitate access for those whom the research ultimately aims to impact (Figure 4). Eventually, an ideal framework would involve bidirectional collaboration between community members and academic researchers, where the community could not only access interpretable versions of study findings but also inform the direction of future research.

There are several limitations to this study. First, the algorithm describes severity categories that are not congruent with the clinical standards for classification through complicated and uncomplicated diverticulitis. The Hinchey system is widely used for this purpose, which was originally described in 1978 and subsequently modified with the onset of computed tomography to facilitate image-based staging.^50^^,^^51^ Complicated diverticulitis is often defined through a modified Hinchey stage >1a which encompasses pericolic abscesses, intraabdominal abscesses, purulent peritonitis, and feculent peritonitis.^52^ Although not explicitly addressed by the Hinchey criteria, other findings indicative of complicated diverticulitis include fistulas, obstructions, or strictures.^52^ While our intention was to approximate gradations of disease severity, the algorithm must be modified for future studies wishing to isolate complicated diverticulitis. Second, algorithm validation via manual chart review was performed in an independent institution due to lack of access to clinic notes in the *All of Us* Research Program. Thus, the reported performance may not reflect the algorithm’s performance in the *All of Us* Research program itself and is limited by variability across reviewers. Third, we required participants to complete their initial survey within 5 years of diverticular disease diagnosis. Given that social needs are often transitory in nature, the barriers reported at the time of survey completion may not reflect the status at the time of the clinical episode for diverticular disease. This is compounded by variability in dates when surveys were completed in the *All of Us* Research Program, and future work is needed for best practices when collating social variables obtained at different time points in the *All of Us* Research Program. Fourth, the Brokamp Deprivation Index was pre-computed using 3-digit zone improvement plan codes, which may not translate to census tract calculations or to other area-level metrics. Lastly, important constructs such as rurality, family obligations, and perceived healthcare discrimination were not examined as part of this study.

## Conclusion

By use of a defined phenotyping algorithm for diverticular disease severity, unmet social needs were found to be associated with greater severity of diverticulitis presentations.

## Supplementary Material

ocae238_Supplementary_Data

## Data Availability

Data and code for the analysis in the *All of Us* Research Program was performed in the Researcher Workbench workspace title “Diverticular Disease Phenotyping and Social Determinants of Health.” This workspace may be shared upon request with approved researchers on the Researcher Workbench who have completed privacy compliance training. Additional description of the phenotyping algorithm is available on the Phenotype Knowledge Base entry titled “Diverticular Disease Severity, Left Colonic.” To protect the privacy of individuals who participated in the study, data from Vanderbilt’s Synthetic Derivative is unavailable for public sharing but code may be shared upon reasonable request.
