# Supplementary material for: Unmet social needs and diverticulitis: a phenotyping algorithm and cross-sectional analysis
Source: J Am Med Inform Assoc. 2025 Mar 14;32(5):866–75. doi: 10.1093/jamia/ocae238 (PMC12012367; doi:10.1093/jamia/ocae238)
Supplement: ocae238_Supplementary_Data [file ocae238_supplementary_data.docx]

**Supplementary Material**

### Phenotyping Algorithm

When using International Classification of Diseases, 10^th^ revision (ICD-10) codes to identify complications of diverticular disease, performance has previously been previously reported to be highly variable.[1] To investigate this further, we extracted 150 cases of patients who were assigned complicated diverticulitis codes and manually reviewed the charts to determine whether these patients met criteria for complicated diverticulitis as specified in the American Society of Colon and Rectal Surgeons Clinical Practice Guidelines.[2] Overall, 70% of those who were assigned a complicated diverticulitis code had complicated diverticulitis confirmed on manual review. The most common misassignment reason was for a contained microperforation (62%). (Supplementary Table 1)

| Supplementary Table 1: Manual review of patients with complicated diverticulitis codes | |
| --- | --- |
| **Misassignment reason** | **Count** |
| Microperforation | 28 |
| Suspected diagnosis but not confirmed on imaging or scope | 8 |
| Other or unclear | 5 |
| Recurrent uncomplicated disease | 4 |
| ICD-10 codes used for complicated diverticulitis: K57.20, K57.21, K57.40, K57.41, K57.80, K57.81 | |

Our rule-based algorithm required a diagnostic code within 7 days after an intestinal imaging study or endoscopic procedure, which aimed to capture disease confirmed by visualization. Similarly, we required controls to have both absence of diverticular disease diagnostic codes and an endoscopic lower gastrointestinal procedure to reduce the likelihood that asymptomatic disease would be captured in our control group.

Notably, our algorithm deviates from the clinical standard of classifying patients according to uncomplicated versus complicated disease. Because of this, we quantified how complications were distributed across the groups presented in the primary manuscript. For the Operative or Recurrent Inpatient Diverticulitis group, 89% of the operative cases had confirmation of complicated disease on manual review as opposed to 37% of the recurrent inpatient cases. When comparing the Mild Diverticulitis Group to the Operative or Recurrent Inpatient Diverticulitis group, abscess was the most common complication and all complications occurred at greater frequency in the Operative or Recurrent Inpatient Diverticulitis group (Supplementary Table 2). For future studies interested in isolating complicated diverticulitis, we recommend limiting inclusion to operative cases. Within operative cases, a further distinction may be necessary between elective colectomy for recurrent uncomplicated diverticulitis and procedures performed for complications on an urgent basis. Use of natural language processing on image or procedure notes would likely enhance algorithm performance.

| Supplementary Table 2: Counts of complications by sub-phenotype groups | | |
| --- | --- | --- |
| **Complication** | **Mild Diverticulitis** | **Operative or Recurrent Inpatient Diverticulitis** |
| Macroperforation | 5 | 43 |
| Fistula | 1 | 27 |
| Abscess | 7 | 64 |
| Stricture | 1 | 5 |
| Unclear or not specified | 11 | 12 |
| Obstruction | 0 | 3 |
| Values represent patient counts | | |

We also considered two algorithm modifications. In the first, we increased the temporal window for case inclusion from 7 to 30 days. This did not substantially alter case inclusion counts (<1% change). In the second, we required an antibiotic prescription for inclusion in the Mild Diverticulitis group. This was abandoned after exploratory manual chart review identified a high frequency of false negatives, where clinical notes explicitly mentioned antibiotic prescriptions that were not identified on extraction. Further, with newer guidelines recommending caution for antibiotics in the context of acute uncomplicated diverticulitis, we believed the requirement of an antibiotic prescription would limit future generalizability.[2] Additional details about code inclusions can be found on the page in the Phenotype Knowledge Base.[3]

There are many limitations of this algorithm. The most common reasons for misassignment were an inability to incorporate information mentioned in outside hospital notes (37%), counting admissions where the primary reason was for another disease (25%), or incongruence between the diagnostic code and imaging or procedure reports (16%). Further, our requirement of imaging or procedure reports in close proximity to a diverticular disease diagnostic code excludes cases with these studies performed at other hospital systems. At our institution, we identified and reviewed 959 patients who had been assigned an ICD-9 or ICD-10 code for diverticular disease but did not qualify as a case according to our algorithm. From these, 202 (21%) should have qualified as a case based on manual review. Studies with greater tolerance for false positives may consider relaxation of the image- or procedure- based requirements presented here.

### Variable Transformations in the *All of Us* Research Program

Selected survey items and methodologies for transforming responses are described in Supplemental Tables 3 – 11 and grouped by Healthy People 2030 domains.[4] Answer responses of “don’t know”, “skip”, or “prefer not to answer” were collapsed to “Skip or PNA”. When applicable, survey responses were scored according to the original source.

| Supplementary Table 3: Variable Transformations in the Economic Stability domain | | | |
| --- | --- | --- | --- |
| **Variable Description** | **All of Us Codebook Variable (survey)** | **Original Levels** | **New Levels** |
| Annual income | Income_annual_income (Basics Survey) | more 200k  150k-200k  100k-150k  75k-100k  50k-75k  35k-50k  25k-35k  10k-25k  Less 10k  PMI: Prefer not to answer  PMI: Skip | more 100k  50-100k  less 50k  Skip or PNA |
| Employment status | Employment_employmentstatus (Basics Survey) | Employed for wages  Self-employed  Out of work for 1 year or more  Out of work for less than 1 year  Homemaker  Student  Retired  Unable to Work  Prefer Not to Answer | Employed  Out of work  Student, homemaker, or retired  Unable or PNA |
| Stable housing concern | Livingsituation_stablehouseconcern (Basics Survey) | Yes  No |  |

| Supplementary Table 4: Questionnaires in the Economic Stability domain | | |
| --- | --- | --- |
| **Questionnaire** | **All of Us Codebook Variable** | **Scoring** |
| Hunger Vital Sign | hvs_1  hvs_2 | 1. answer that either or both of the two statements is ‘often true’ or ‘sometimes true’ (vs. ‘never true’) |

| Supplementary Table 5: Variable Transformations in the Education Access and Quality domain | | | |
| --- | --- | --- | --- |
| **Variable Description** | **All of Us Codebook Variable** | **Original Levels** | **New Levels** |
| Highest grade of education | Educationlevel_highest_grade (Basics Survey) | Advanced Degree  College Graduate  College One to Three  Twelve or GED  Nine Through Eleven  Five Through Eight  One Through Four  Never Attended  PMI: Prefer Not To Answer  PMI: Skip | College or Advanced Degree  High School Degree or Equivalent  Less than High School Degree or Equivalent  Skip or PNA |

| Supplementary Table 6: Variable Transformations in the Healthcare Access and Quality domain | | | |
| --- | --- | --- | --- |
| **Variable Description** | **All of Us Codebook Variable** | **Original Levels** | **New Levels** |
| Current health insurance coverage | Insurance_healthinsurance (Basics Survey) | Yes  No  Prefer not to answer  Don’t know | Yes  No  Skip or PNA |
| Insurance not accepted in last 12 months | insurance_insuranceaccepted (Healthcare Access and Utilization Survey) | Yes  No  Don’t know |  |
| Delayed getting medical care due to copay | delayedmedicalcare_cantaffordcopay (Healthcare Access and Utilization Survey) | Yes  No  Don’t know |  |
| Delayed getting medical care due to deductible | delayedmedicalcare_deductibletoohigh (Healthcare Access and Utilization Survey) | Yes  No  Don’t know |  |
| Delayed getting medical care due to having to pay out of pocket | delayedmedicalcare_hadtopayoutofpocket (Healthcare Access and Utilization Survey) | Yes  No  Don’t know |  |
| Delayed getting medical care due to transportation | delayedmedicalcare_transportation (Healthcare Access and Utilization Survey) | Yes  No  Don’t know |  |
| Needed prescription medicine but couldn’t afford it | cantaffordcare_prescriptionmedicines (Healthcare Access and Utilization Survey) | Yes  No  Don’t know |  |
| Needed emergency care but couldn’t afford it | Cantaffordcare_emergencycare (Healthcare Access and Utilization Survey) | Yes  No  Don’t know |  |
| Needed specialist care but couldn’t afford it | cantaffordcare_specialist (Healthcare Access and Utilization Survey) | Yes  No  Don’t know |  |
| Needed follow up care but couldn’t afford it | cantaffordcare_followupcare (Healthcare Access and Utilization Survey) | Yes  No  Don’t know |  |

| Supplementary Table 7: Questionnaires in the Healthcare Access and Quality domain | | |
| --- | --- | --- |
| **Questionnaire** | **All of Us Codebook Variable** | **Scoring** |
| Brief Health Literacy Screen | overallhealth_medicalformconfidence  overallhealth_healthmaterialassistance  overallhealth_difficultunderstandinfo | 1. Reverse score the item addressing confidence to forms 2. Sum the 1-5 scores (range 3-15) |

| Supplementary Table 8: Variable Transformations in the Social and Community Context domain | | | |
| --- | --- | --- | --- |
| **Variable Description** | **All of Us Codebook Variable(s)** | **Original Levels** | **New Levels** |
| Speaking non-English language at home | chis_1 (Social Determinants of Health Survey) | Yes  No  Prefer not to answer |  |
| English proficiency | chis_1_xx (Social Determinants of Health Survey) | Very well  Well  Not well  Not at all  Prefer not to answer  Don’t know | Well or very well  Not well or not at all  Don’t know or PNA |

| Supplementary Table 9: Questionnaires in the Social and Community Context domain | | |
| --- | --- | --- |
| **Questionnaire** | **All of Us Codebook Variable** | **Scoring** |
| Modified Medical Outcomes Study Social Support Survey | sdoh_mos_ss_1  sdoh_mos_ss_2  sdoh_mos_ss_3  sdoh_mos_ss_4  sdoh_mos_ss_5  sdoh_mos_ss_6  sdoh_mos_ss_7  sdoh_mos_ss_8 | 1. Rate item on 5-point scale (1 None of the time, 5 All of the time) 2. Average of all 8 items 3. Must have at least 6 items present to compute average |

| Supplementary Table 10: Variable Transformations in the Neighborhood and Built Environment domain | | | |
| --- | --- | --- | --- |
| **Variable Description** | **All of Us Codebook Variable(s)** | **Original Levels** | **New Levels** |
| Problems with any of the following at the place you live (housing quality) | ahc_2 (Social Determinants of Health Survey) | Bug infestation  Mold  Lead paint or pipes  Inadequate heat  Oven or stove not working  No or not working smoke detector  Water leaks  None of the above | Any  None |

| Supplementary Table 11: Questionnaires in the Neighborhood and Built Environment domain | | |
| --- | --- | --- |
| **Questionnaire** | **All of Us Codebook Variable(s)** | **Scoring** |
| Neighborhood Physical Disorder Scale | nds_1 (Social Determinants of Health Survey)  nds_2  nds_3  nds_4  nds_5  nds_6 | 1. Reverse score two items (nds_5, nds_6) 2. Assign 4 point agreement scale (1 strongly disagree 4 strongly agree) 3. Take average of items 4. Set to missing if any of the items are NA |

For the Brokamp Deprivation index, we created binary indicators of individual social barriers from the extracted survey information. These included annual houshold income less than $50,000, reporting “out of work” on the employment question, education less than high school or equivalent, any of the delayed or can’t afford care items, no insurance or reporting that insurance was not accepted, housing instability, housing quality concern, or an English proficiency barrier. Surveys were converted to top box scoring as described in the primary manuscript for the Hunger Vital Sign, Brief Health Literacy Screen, Perceived Neighborhood Physical Disorder Scale, and the Modified Medical Outcomes Study Social Support Survey. Reporting a non-top box score was considered a social barrier.

For the adjusted analysis, an unmet social need was identified if the following barriers were reported: positive Hunger Vital Sign screen, any delayed or can’t afford care item, or housing instability. For a participant to be coded as no unmet social need, they must have had available entries indicating no need for at least two of the three barriers.

### Inclusion Flowchart


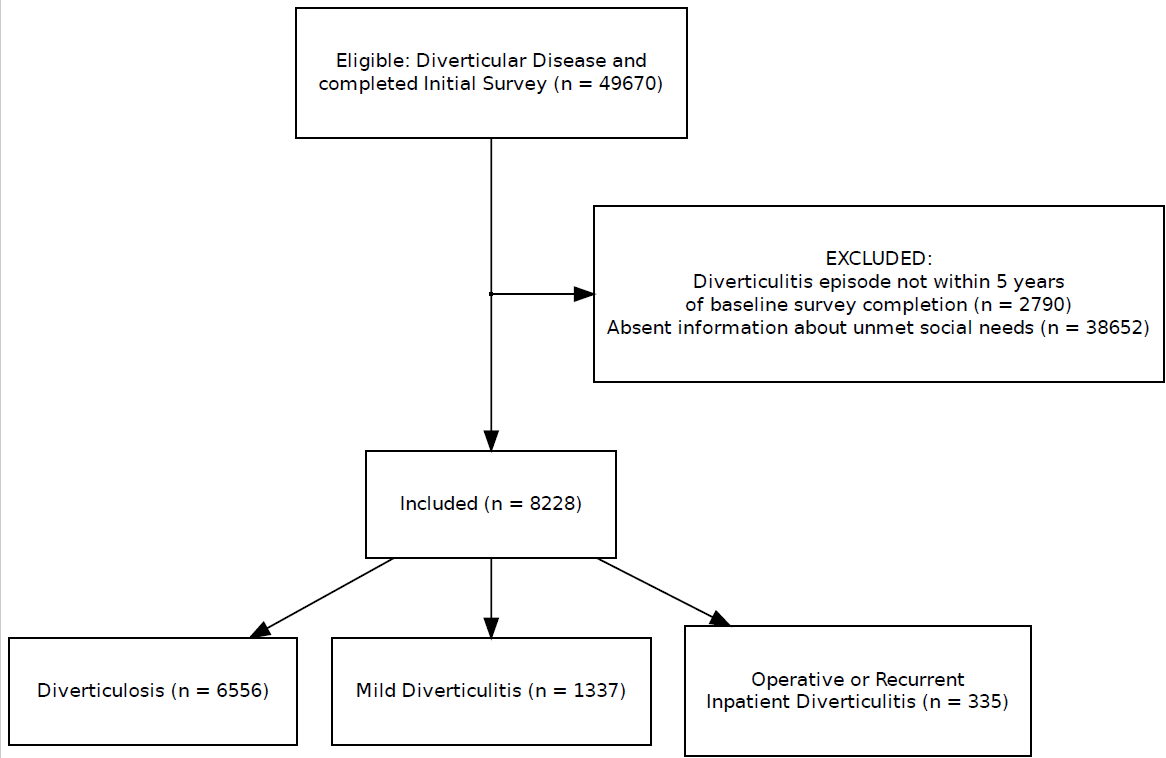


*Supplementary Figure 1: Inclusion Flowchart. Participants were identified from the All of Us v7 Controlled Tier dataset. They were excluded if the diverticular disease inclusion date was not within 5 years of earliest survey completion. Patients with missing information about the unmet social needs examined in the adjusted analysis (food insecurity, housing instability, delayed or can’t afford care) were also excluded.*

### Univariable Comparison Sensitivity Analysis

The primary manuscript examined the distribution of social barriers across three groups: “Diverticulosis”, “Mild Diverticulitis”, and “Operative or Recurrent Inpatient Diverticulitis”. We also examined the distribution in the “Operative or Recurrent Inpatient Diverticulitis” relative to an age- and sex at birth-matched group of controls without diverticular disease. (Supplementary Table 12).

| Supplementary Table 12: Characteristics of Operative or Recurrent Inpatient Diverticulitis relative to age- and sex-matched controls | | | |
| --- | --- | --- | --- |
| **Characteristic** | **Age- and sex- matched controls**  (N = 330) | **Operative or Recurrent Inpatient Diverticulitis**  (N = 330) | **p-value** |
| Sex at birth |  |  | >0.90 |
| Female | 211 (64%) | 211 (64%) |  |
| Male | <120 | <120 |  |
| Other | <20 | <20 |  |
| Age at CDR cutoff (years) | 66 (58, 72) | 66 (58, 72) | >0.90 |
| BMI (kg/m2) | 26 (23, 30) | 30 (27, 35) | <0.01 |
| Charlson comorbidity index | 2 (1, 4) | 4 (2, 6) | <0.01 |
| Annual income |  |  | <0.01 |
| Less than 50k | 87 (30%) | 120 (44%) |  |
| 50k-100k | 75 (26%) | 70 (26%) |  |
| More than 100K | 125 (44%) | 82 (30%) |  |
| Employment |  |  | 0.30 |
| Out of Work | <20 | <20 |  |
| Employed | 153 (47%) | 133 (41%) |  |
| Other | <160 | <180 |  |
| Hunger Vital Sign positive screen | 24 (13%) | 32 (20%) | 0.11 |
| Highest education level |  |  | <0.01 |
| Less than High School Degree or Equivalent | <20 | 23 (7.1%) |  |
| High School Degree or Equivalent | 27 (8.3%) | 46 (14%) |  |
| Some College, College degree, or Advanced degree | 297 (91%) | 256 (79%) |  |
| Insurance: none or not accepted | 60 (18%) | 24 (7.3%) | <0.01 |
| Brief health literacy screen | 15 (14, 15) | 15 (13, 15) | 0.02 |
| Delayed or can't afford care | 92 (30%) | 78 (30%) | >0.90 |
| Housing quality concern | 33 (19%) | 28 (19%) | >0.90 |
| Housing instability | 45 (14%) | 88 (27%) | <0.01 |
| Perceived Physical Neighborhood Disorder Scale | 1.67 (1.33, 2.17) | 1.50 (1.00, 1.83) | <0.01 |
| Modified Medical Outcomes Study Social Support Survey | 3.87 (2.88, 4.64) | 4.00 (3.00, 4.88) | 0.30 |
| English proficiency barrier | <20 | <20 | 0.10 |
| PROMIS Global Mental score (T-score) | 51 (44, 56) | 48 (41, 53) | <0.01 |
| PROMIS Global Physical score (T-score) | 51 (45, 58) | 45 (37, 51) | <0.01 |
| Unmet Social Need *^1^* | 125 (38%) | 167 (51%) | <0.01 |
| Values represent n (%) or Median (IQR). Missingness counts omitted and values less than or equal to 20 are not shown to maintain compliance with All of Us Data and Statistics Dissemination Policy. P values represent Wilcoxon rank sum test; Pearson’s Chi-squared test  CDR: Curated Data Repository. PROMIS: Patient-Reported Outcomes Measurement Information System  *^1^* Unmet social need is a composite variable representing food insecurity, housing instability, and delayed or can’t afford care items | | | |

### Adjusted Modeling Sensitivity Analyses

The adjusted model focused on three social needs: food insecurity, housing instability, and delaying or inability to afford care. These needs were obtained from three surveys (Basics, Healthcare Access and Utilization, Social Determinants of Health). In the *All of Us* Research Program, not all surveys were completed at the same time. Supplementary Figure 2 demonstrates variability in time between survey completion. Relative to the earliest survey, the months before completion of subsequent surveys was a mean [standard deviation] of 7.7 [9.4] months for the Healthcare Access and Utilization Survey and 23.6 [15.7] months for the Social Determinants of Health Survey.


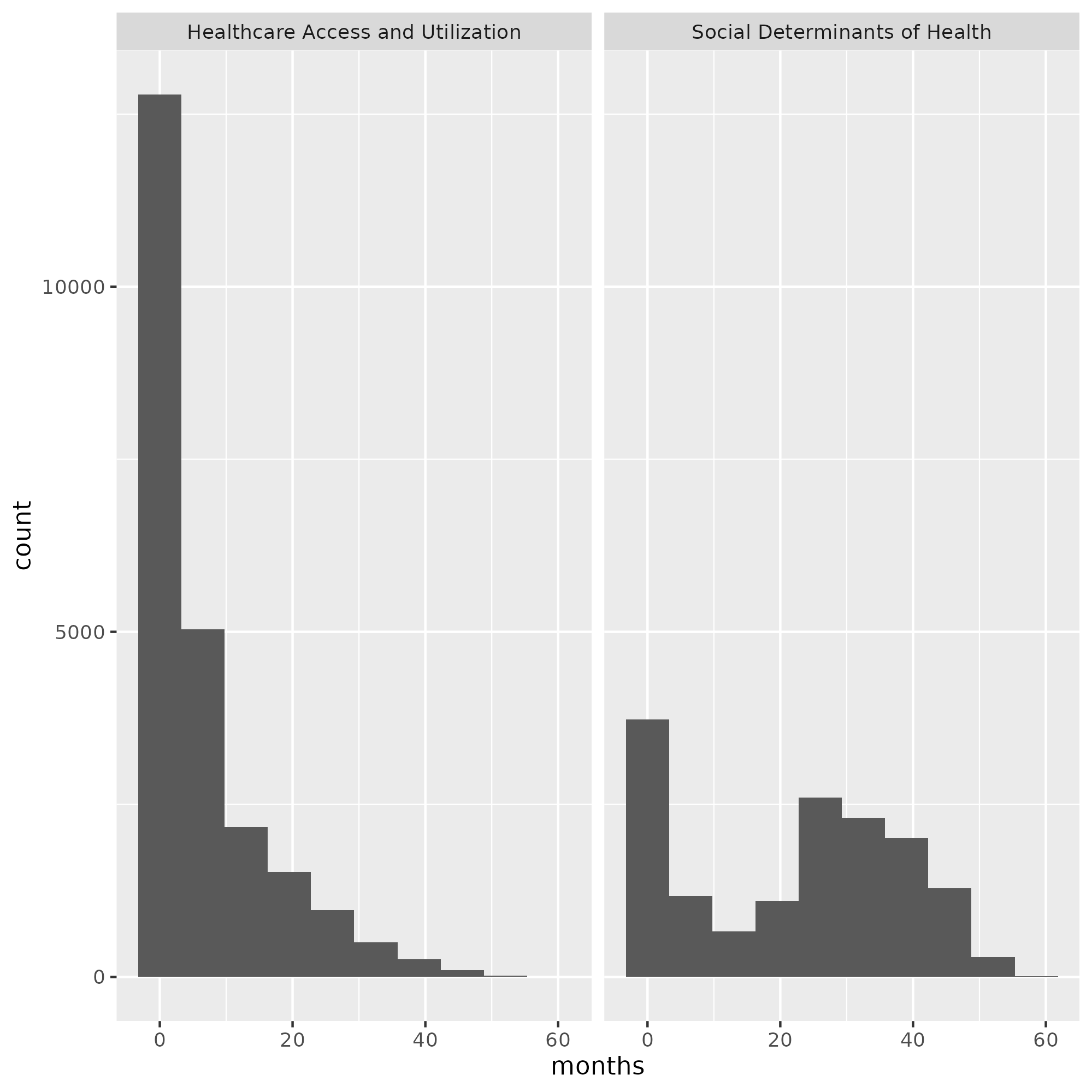


Supplemental Figure 2: Histograms of the difference in months between completion of the earliest survey and completion of the Healthcare Access and Utilization Survey and the Social Determinants of Health Survey for the cohort.

For the primary manuscript, we included only participants with a time difference of 5 years between the earliest survey and the diverticular disease inclusion date. Sensitivity analyses presented in Supplementary Table 13 perform the adjusted analysis when limiting the inclusion time difference to 2.5 years and when considering alternative surveys as the inclusion survey. An unmet social need remained associated with more severe disease presentations when limiting the time difference to 2.5 years between inclusion survey completion and procedure date. This retained significance when changing the inclusion survey to Healthcare Access and Utilization, but not when changing the inclusion survey to Social Determinants of Health.

| Supplementary Table 13: Adjusted Modeling Sensitivity Analyses | | | | | | | | | |
| --- | --- | --- | --- | --- | --- | --- | --- | --- | --- |
| **Characteristic** | Survey-inclusion date window narrowed to 2.5 years | | | Inclusion survey: Healthcare Access and Utilization | | | Inclusion survey: Social Determinants of Health | | |
|  | **OR** | **95% CI** | **p-value** | **OR** | **95% CI** | **p-value** | **OR** | **95% CI** | **p-value** |
| Age at CDR cutoff (years) | 0.98 | 0.97, 0.99 | <0.01 | 0.98 | 0.97, 1.00 | 0.02 | 0.98 | 0.96, 1.00 | 0.08 |
| Sex at birth: Female | 1.27 | 0.96, 1.68 | 0.10 | 1.53 | 1.13, 2.11 | <0.01 | 1.43 | 0.91, 2.29 | 0.12 |
| BMI (kg/m2) | 1.00 | 0.98, 1.02 | >0.90 | 1.00 | 0.98, 1.02 | >0.90 | 1.02 | 0.99, 1.05 | 0.20 |
| Charlson Comorbidity Index | 1.07 | 1.02, 1.12 | <0.01 | 1.08 | 1.03, 1.14 | <0.01 | 1.08 | 0.99, 1.16 | 0.08 |
| Unmet social need | 1.39 | 1.03, 1.88 | 0.03 | 1.40 | 1.01, 1.94 | 0.04 | 1.52 | 0.93, 2.44 | 0.09 |
| Insurance: None or not accepted | 0.61 | 0.34, 1.03 | 0.09 | 0.83 | 0.47, 1.38 | 0.50 | 1.05 | 0.47, 2.06 | >0.90 |
| Annual income: Less than 50k | 1.02 | 0.75, 1.37 | 0.90 | 0.97 | 0.69, 1.35 | 0.90 | 0.85 | 0.50, 1.40 | 0.60 |
| Education: Less than High School | 0.96 | 0.54, 1.60 | 0.90 | NA | NA | NA | NA | NA | NA |
| OR: Odds Ratio. CI: Confidence Interval. CDR: Curated Data Repository. BMI: Body Mass Index. 50k: $50,000. NA: Not available as this variable was removed from the model with the non-zero variance filter step of data processing. | | | | | | | | | |

### References

1 Erichsen R, Strate L, Sørensen HT, *et al.* Positive predictive values of the International Classification of Disease, 10th edition diagnoses codes for diverticular disease in the Danish National Registry of Patients. *Clin Exp Gastroenterol*. 2010;3:139–42. doi: 10.2147/CEG.S13293

2 Hall J, Hardiman K, Lee S, *et al.* The American Society of Colon and Rectal Surgeons Clinical Practice Guidelines for the Treatment of Left-Sided Colonic Diverticulitis. *Diseases of the Colon & Rectum*. 2020;63:728–47. doi: 10.1097/DCR.0000000000001679

3 Ueland T, Niu X. Diverticular Disease Severity, Colonic. PheKB. Vanderbilt University School of Medicine. 2024. https://phekb.org/phenotype/diverticular-disease-severity-colonic (accessed 8 January 2023)

4 Tesfaye S, Cronin R, Lopez-Class M, *et al.* Measuring Social Determinants of Health in the All of Us Research Program: Technical Document. 2023;2023.06.01.23290404.
